# Optimization of the Oxygen Permeability of Non-Silicone Hydrogel Contact Lenses Through Crosslinking Modifications

**DOI:** 10.3390/gels10110726

**Published:** 2024-11-09

**Authors:** Clara Lim, María García-Montero, Andrew Courtis, Paul Hainey, David Madrid-Costa, Almudena Crooke

**Affiliations:** 1 Department of Optometry and Vision, Faculty of Optics and Optometry, Complutense University of Madrid, 28037 Madrid, Spainmgarc01@ucm.es (M.G.-M.); damadrid@ucm.es (D.M.-C.); 2 Department of Biochemistry and Molecular Biology, Faculty of Optics and Optometry, Complutense University of Madrid, 28037 Madrid, Spain; 3 Research and Development Group, Mark’ennovy, Widnes WA8 0RP, UK; andrew.courtis@gmail.com (A.C.); paul.hainey@markennovy.com (P.H.); 4 Clinical and Experimental Eye Research Group, UCM 971009, Faculty of Optics and Optometry, Complutense University of Madrid, 28037 Madrid, Spain

**Keywords:** crosslinking, hydrogel, oxygen permeability, soft contact lens

## Abstract

The main weakness of non-silicone hydrogel contact lenses is their low oxygen permeability (Dk). Hence, we have tried to optimize their Dk using various concentrations and lengths of the poly (ethylene glycol) dimethacrylate crosslinker in a mixture of N,N-Dimethylacrylamide and Cyclohexyl methacrylate monomers. After synthesizing the different contact lenses, we evaluated their chemical, optical, and mechanical properties. The resultant non-silicone hydrogel contact lenses presented similar high water contents (75.69–80.60%) and adequate optical (e.g., a transmittance ranging from 85.91% to 99.91% and a refractive index between 1.3630 and 1.3740) and elongation at break (178.95–356.05%) characteristics for clinical applications. Conversely, they presented high contact angles (81.00–100.00°) and a low Young’s modulus (0.066–0.167 MPa). Regarding the impact of the crosslinking modifications, the water content, contact angle, refractive index, transmittance, and Young’s modulus of the synthesized lenses were slightly affected by crosslinker conditions. In contrast, the elongation at break (178.95–356.05%) and, more importantly, the oxygen permeability, which reached values of up to 73.90 Fatt units, were considerably impacted by the crosslinker conditions. To our knowledge, this study demonstrates for the first time that, in addition to water, other usual hydrogel components, like crosslinkers, can modulate the Dk of non-silicone contact lenses. It also provides a simple and scalable method to fabricate more permeable non-silicone lenses.

## 1. Introduction

Since their creation, contact lenses (CLs) have evolved in terms of material, fabrication process, and optical design, which have extended their application possibilities, ranging from vision correction to aesthetic and therapeutic uses [1,2,3]. In particular, the most frequently used CL materials are soft CLs [2], with a global market size of USD 9.5 billion in 2022 [4]. 

Soft CLs are made of either hydrogel (Hy) or silicone hydrogel (SiHy), offering numerous advantages such as enhanced comfort and compatibility with the eye due to their high water content [5]. SiHy CLs provide superior oxygen permeability (up to 140 Fatt units) to the eye thanks to their silicon–oxygen (Si–O) structural bond [2,6]. Maintaining adequate oxygen levels in the cornea is essential to prevent one of the most common complications associated with soft CLs: corneal hypoxia–oxygen deprivation of the cornea, which can manifest as red eyes, corneal swelling, and corneal vascularization [3,7,8,9,10,11,12,13]. The introduction of SiHy CLs has been instrumental in addressing a significant portion of this issue [5,14]. However, they have not fully resolved many other challenges CL wearers face. Indeed, the presence of silicone also has drawbacks as it enhances the hydrophobicity and rigidity of the hydrogel, as well as lipid deposition [15], which promotes inflammation, discomfort, and dryness, which are the reasons wearers discontinue CLs [16,17]. In contrast, Hy CLs present a lower oxygen permeability (below 60 Fatt units) [6], facilitating corneal infection and, thus, corneal infiltration events [18]. 

In this context, there have been fewer efforts to improve the properties of Hy CLs than SiHy CLs. Thus, several authors have evaluated the possibility of enhancing oxygen’s passage through SiHy CLs by changes in their components (e.g., silicone monomers [19] or particles [20,21], plasticizers [22], and crosslinkers [23,24]). Regarding Hy CLs, the literature links oxygen passing through them to their water content (WC). Hence, to calculate their Dk based on lens WC, several equations have been proposed [25,26] and applied in several works on CLs [11,27,28,29,30,31,32,33,34]. Nevertheless, the ISO standards do not recommend using these equations [35]. In alignment with these recommendations, Chirila [36] asserted that it is essential to determine experimentally the oxygen permeability because those equations focus only on synthetic hydrogels commonly employed in silicone-free CL manufacturing.

In the present work, we aim to optimize the oxygen permeability (Dk) of non-silicone hydrogel CLs, using a component commonly used in the CL industry to maintain the cohesiveness of the polymer network, the crosslinker agent. Several studies have highlighted the crosslinker´s impact on the properties of hydrogels [37]. Hence, we have evaluated its ability to modulate oxygen permeability and other chemical, optical, and mechanical properties of non-silicone hydrogel CLs. More specifically, we have studied the impact of various lengths and concentrations of the ethylene glycol dimethacrylate crosslinker on a mixture of N,N-Dimethylacrylamide, and Cyclohexyl methacrylate monomers. This work highlights the strong effect of crosslinker modifications on the oxygen permeability of non-silicone CLs. To our knowledge, it demonstrates for the first time that, in addition to water, other usual hydrogel components, like crosslinkers, can modulate the Dk of non-silicone contact lenses. Furthermore, it provides a simple and scalable method to fabricate more permeable non-silicone lenses.

## 2. Results and Discussion

We analyze the chemical, optical, and mechanical properties of the CLs obtained from the seven manufactured polymer rods (i.e., sample 1E, sample 3E-A, sample 3E-B, sample 3E-C, sample 3E-D, sample 9E, and sample 23E).

### 2.1. Water Content Measurement Results

We show the water contents (WCs) of the fabricated samples in Table 1. 

All samples presented a WC higher than 50% (WC values ranged between 75.69% and 80.60%) (Table 1). Thus, they may be considered high-WC CLs based on the ISO standard 18369-1:2017 [38]. The 3-EGDMA concentration affected the CL WC, reducing its content by almost 5% with increasing concentration (Table 1; samples 3E-A, 3E-B, 3E-C, and 3E-D). Regarding crosslinker length and for a similar crosslinker concentration (mol %), the WC increased from 1-EGDMA to 3-EGDMA but decreased with 9-EGDMA and 23-EGDMA (Table 1).

The WC is a pivotal characteristic of soft CLs [39,40]. Various studies have proven its effect on CL comfort by modifying properties like the modulus and wetting characteristics, as well as oxygen permeability [39,41]. The high values of the WC obtained in our work are due to the high amount of the hydrophilic monomer DMAA used for CL synthesis [22,42,43]. The slight decrease in CL-WC observed in our study related to the increased concentration of crosslinkers is consistent with the findings of other authors [24]. For instance, in the study of Mohammed et al. [24], an increase in the percentage of the 1-EGDMA crosslinker from 0 to 2 wt% resulted in a decrease in the WC by 7 %. This effect may occur because increasing the concentration of the crosslinking agent increases the structural restriction of the polymer chains and, thus, the inside free volume decreases, which, in turn, hinders the entry of water through the network and its swelling. 

### 2.2. Oxygen Permeability Measurement Results

To validate the Dk measurement method, we evaluated the permeability of the Comfi Daily disposable Etafilcon A-based CL. This lens presented a Dk of 17.4 ± 0.1 Fatt units, a value close to that calculated using the Morgan and Efron (M&E) [25] equation with the 1 Day Acuvue (Vistakon) CL (Dk = 18.1 Fatt units), a lens made of the same material.

After that, we measured the oxygen permeability (Dk) of the samples synthesized and compared their values with the ones obtained from the M&E [25] and Young and Benjamin (Y&B) [26] equations, which assume that the Dk is exclusively dependent on the WC.

**Figure 1:**
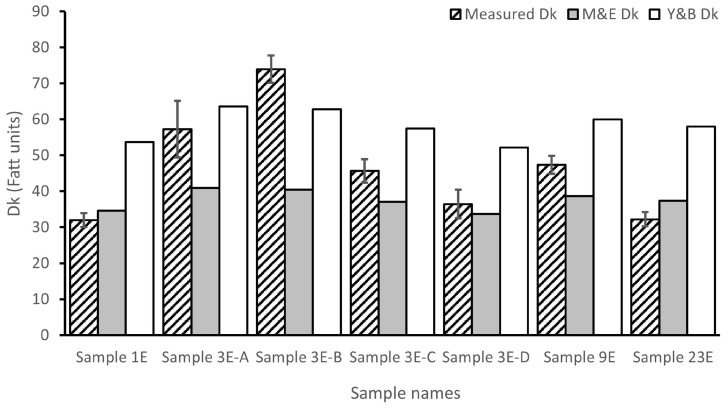
Experimental and theoretical oxygen permeability (DK) of the contact lenses synthesized. Experimental Dk data are shown as means ± standard deviation. Morgan and Efron (M&E) [25] and Young and Benjamin (Y&B) [26] equations were employed to obtain the theoretical Dk values.

The measured Dk of the samples varies from 31.95 to 73.90 Fatt units (Figure 1).

The Dk parameter evaluates the oxygen diffusion through a material, with D being the diffusivity and k being the solubility of the material [44]. It is critical in the case of CL that the material allows the oxygen molecule to reach the eye tissue, thus avoiding hypoxia [45]. Consequently, multiple works have attempted to expand the knowledge of oxygen permeation mechanisms through CL and improve the material’s Dk [1]. 

The oxygen diffusion path through the CLs depends on the hydrogel material. While in SiHy CLs, the oxygen flows through the hydrophobic phase, in Hy ones, the diffusion goes through the hydrophilic phase [23,26,46]. This difference in the mechanism of oxygen passage explains the Dk diversity between the various materials of hydrogel CLs. Hence, while commercial SiHy CLs present Dk values ranging from 60 to above 100 Fatt units, Hy CLs show lower oxygen permeability with Dk values below 60 Fatt units [6,47]. In our study, six of the seven non-silicone hydrogel CLs fabricated presented Dk values like those of commercial Hy CLs (Figure 1). Nevertheless, sample 3E-B presented a high Dk value (73.90 Fatt units) like that of commercial SiHy CLs (Figure 1). This result and all the Dks obtained for the total fabricated samples show that the crosslinker concentration (see the Dk of samples 3E-A to 3E-D) and its length (see the Dk of samples 1E, 3E-B, 9E, and 23E) have an impact on oxygen permeability (Figure 1).

Other authors have studied the impact of the 1-EGDMA concentration on SiHy CL oxygen permeability. For instance, Mohammed et al. [24] showed that the Dk values of SiHy CLs could decrease from 58.9 to 53.5 Fatt units when the 1-EGDMA content increased from zero to two wt%. Similarly, Wu et al. [23] observed that increasing concentrations of 1-EGDMA (from 0.5 to 1 wt%) decreased the Dk value of hydrogels. 

In our study, we also observed a decrease in the Dk with the rise of the concentration of the 3-EGDMA crosslinker from 0.27 to 0.99 wt% (Figure 1; samples 3E-B, 3E-C, and 3E-D). Similarly to the previous studies, as the crosslinker concentration increases, the space between the monomer chains is reduced, limiting oxygen passage. Surprisingly, the Dk rose when the 3-EGDMA concentration went from 0.15 to 0.27 wt% (Figure 1; samples 3E-A and 3E-B). This fact might be due to an increase in the entanglement of the polymer chains, which can happen with lowly crosslinked hydrogels [48,49,50,51]. Thus, in our study with a lengthier EGDMA version, the oxygen permeability does not vary linearly with the crosslinkers´ concentration. 

As shown in Figure 1, some of our CLs presented Dk values below (Sample 1E and Sample 23E) or above (Sample 3E-B) those estimated by the equations of Morgan and Efron [25] and Young and Benjamin [26], despite having a similar WC (Table 2). These results, particularly that of Sample 3E-B (experimental Dk = 73.90 Fatt units versus theoretical Dk (Y&B/M&E) = 62.78/40.45 Fatt units), indicate that it is possible to improve the Dk of Hy CLs by modifying crosslinker conditions even with high WC values (80.28%) (Figure 1 and Table 1). More importantly, they show that water is not the only component that defines the Dk of Hy CLs and opens new possibilities for developing more permeable non-silicone hydrogel CLs.

### 2.3. Contact Angle Measurement Results

We also evaluated the in vitro wettability of the fabricated samples, measuring their contact angles (CAs) (Table 1). All the CLs presented a CA ranging from 81° to 100° (Table 1).

The wettability of a material is the ability of a liquid to spread on the surface of the material. In the case of CLs, increased wettability means a better spread of the tear film over the lens, which is essential for maintaining a stable and uniform tear film on the lens, maintaining ocular health, and enhancing user comfort with reduced friction [52,53]. Although good wettability implies low CA values (i.e., below 90°), there are commercial CLs with CAs above 90 degrees, such as Acuvue Oasys^®^ (Senofilcon A) [54] and PureVision^®^ (Balafilcon A) [55] SiHy CLs. 

Three of our seven samples presented a CA below 90° but above 80°, a surprising result due to their high WC (Table 1). Other authors have reported the poor wettability of the hydrophilic monomer DMAA in comparison with the monomers N-vinyl pyrrolidone (NVP) and acrylic acid (AA) [43,56]. For instance, Hu et al. [56] reported that a CL based on DMAA presented a CA of 90° and that its change with AA monomer triggered a higher wettability, reducing the CA by 16 degrees. Similarly, Tran et al. [42] observed that a hydrogel CL mixture of 2-hydroxyethyl methacrylate (HEMA), NVP, and DMAA tended to increase in wettability when the content of NVP increased at the expense of DMAA.

The remaining samples (3E-A, 3E-B, 9E, and 23-E) present contact angles above 90°. Other authors have reported similar contact angles for commercial CLs like Acuvue Oasys^®^ (CA = 96.4°) [54] and PureVision^®^ (CA = 93.6°) [55] with the sessile drop method. Given that this CA measurement method tends to provide higher CA values than other methods like the captative bubble method, additional studies are necessary to confirm that these contact angles translate into low wettability and are not due to the method used for their determination. Regardless, it appears to be necessary to improve the wettability of our CLs to achieve the good wettability values that silicone-free contact lenses often provide. Possible strategies could be plasma treatment [57] or the incorporation of surfactants [58]. 

The crosslinker length did not affect the CA parameter, but its concentration did, as its value decreased with increasing concentration (see CA of samples 3E-A to 3E–D in Table 2). Given that the sessile drop test is known to have a variation ranging from about 5° [59] or more [55,60], this result could be negligible. In this sense, Seo et al. consider variations below 5° in CA to be insignificant [61]. On the other hand, Ju et al. observed that the increase in the poly (ethylene glycol, EG) diacrylate (PEGDA) crosslinker triggered an augmentation of CA due to the reduction in the WC [62]. Thus, more studies are necessary to confirm our results of the impact of EGDMA concentration and length on the CA. Indeed, some authors have also reported a reduction in CA with an augmentation of the crosslinker length [62,63]. Hence, Askari et al. [63] found that modifying the crosslinkers from 3-EGDMA (0.27 wt%) to a mixture of 3-EGDMA with PEGDA (of either 200 or 600 g/mol) reduced the CA. Similarly, Ju et al. [62] studied the effect of 10, 13, 23, and 45 EG units of PEGDA on CA and found that the longer the EG chain, the higher the wettability. 

### 2.4. Visible Light Transmittance and Transparency Measurment Results

Table 1 shows the values of the light transmittance of the different CLs. All the materials gave high transmittance values, ranging from 85.91 to 99.91%.

The transmittance is a mandatory characteristic of the lens to guarantee vision quality for the wearer and is related to the inner structure of the material [64,65]. Thus, to satisfy visual requirements, hydrogel materials must be able to transmit over 90% of visible light [66], although some commercial CLs present lower transmittance values [67]. 

In our study, all fabricated CLs except sample 3E-D showed a transmittance higher than 90% (Table 1). Sample 3E-D presented a transmittance value slightly below 90% (Table 1), which was similar to the average transmittance values of the commercial Air Optix^®^ (Lotrafilcon A) and PureVision^®^ (Balafilcon A) CLs [67] of 83% and 85%, respectively (between 400 and 700 nm). Consequently, all our lenses presented adequate transparency for CL applications.

The effect of varying crosslinker concentrations on the transmittance did not show a particular pattern. A maximum (99.91%) and minimum (85.91%) transmittance was observed at 0.50 and 0.99 wt% of 3EGDMA, respectively (Table 1; samples 3E-C and 3E-D). Conversely, the number of EG units affected the transmittance, reaching its maximum value (96%) with only one EG unit (Table 1; samples 1E, 3E-B, 9E, and 23E). 

This absence of pattern in transmittance due to crosslinker concentration has also been observed in the work of Seo et al. [61], which employed another crosslinker agent, divinylbenzene. In contrast, Wu et al. [23,24] showed a decrease in transmittance with an increase in the 1-EGDMA concentration from 0.5 to 1.0 wt%. This trend was different from our results and may be due to the shorter version of EGDMA that the authors employed to analyze the crosslinker concentration impact.

### 2.5. Refractive Index Measurement Results

We show the refractive indexes (n) of the samples in Table 2. The CLR 12-70 refractometer could not measure the n of sample 1E. Previous studies have also shown the inability of this refractometer to determine the n of some CLs [68,69]. The other samples presented n values between 1.3630 (sample 3E-A) and 1.3740 (sample 3E-D) (Table 1).

The n is a crucial parameter in the optical design of CLs that determines the CLs’ thickness and curvature. Although the n should be close to that of the cornea [34], which is generally estimated to be close to 1.38 [70], a higher refractive index could be convenient for some designs, such as in high myopia CLs, because it allows for the manufacture of thinner CLs [71]. Moreover, thin lenses provide many advantages, such as more comfort, rapid adaptation, and higher oxygen permeability [72], thus making them more adapted for prolonged CL wear. However, they can be more challenging to handle, more prone to dehydration, and more fragile [72]. Therefore, the n of commercial Hy and SiHy CLs tends to range from 1.37 to 1.44 for Hy, reaching the lowest values with a high WC, and from 1.40 to 1.42 for SiHy [68,69,73,74]. Thus, the n measured in this study falls in the lower range or is slightly lower than the commercialized CLs. For instance, the samples with the highest refractive indexes (sample 3E-D, with *n* = 1.3740, and sample 23E, with *n* = 1.3700) (Table 1) have similar n values to those of the Proclear 1-Day^®^ (Omafilcon A) (*n* = 1.38–1.39 with a 62% WC) [69,73], Acuvue 2^®^ (Etafilcon A) (*n* = 1.40 with a 58% WC) [69,73], and Biotrue ONEday^®^ (Nesofilcon) (*n* = 1.37) CLs, respectively [69].

Moreover, as previous authors have demonstrated [33,68,69,73,74], the n of CLs is inversely related to the WC (Table 1). Thus, both the EGDMA concentration and its length modulate the n, increasing its value when they are increased (Table 1). 

The impact of the concentration of crosslinkers on the n of a hydrogel was studied by Zhou et al. [75]. They synthesized a hydrogel with different concentrations (from five to eleven mol%) of the crosslinker triethylene glycol diacrylate (3-EGDA) and observed its maximum n (*n* = 1.53) at nine mol% 3-EGDA. These authors suggested that a high crosslinker concentration could lead to the inhomogeneity (a high point density) of the CL inner structure and, thus, increase its n [76]. Further studies are needed to confirm the correlation between the n and crosslinker concentration by analyzing the effect of different crosslinking agents and their length.

### 2.6. Tensile Test Measurement Results

To evaluate the possible effect of the crosslinker concentration/length on the mechanical properties of the fabricated samples, we measured the Young´s modulus (E) and elongation at break parameters of the different CLs. 

All the samples had a low Young´s modulus (ranging from 0.066 to 0.167 MPa) and a high elongation at break (ranging from 178.95 to 356.05%) (Table 2). 

These parameters indicate the elasticity and break resistance of the CLs, which are essential to avoid problems on the ocular surface and to make the lens comfortable and handy [77,78,79,80]. Moreover, they allow for the movement of the CL on the eye, permitting tear exchange below the lens [81]. Hence, commercial Hy and SiHy CLs have elastic moduli ranging between 0.3 and 1.9 MPa [78] and elongation at break values ranging from 100% to 250%, according to the measurements of Lonnen et al. [82].

Although our CLs were handled in a laboratory, considering the elasticity of commercial lenses, they had a low elastic modulus (Table 2). On the other hand, their elongation at break values were higher than some commercial lenses, as they could reach higher than 150–200% for the Acuvue2^®^ and 100%–200% for the Air Optix Night and Day^®^ (Lotrafilcon A) CLs [82,83]. Our high elongation at break values could be due to the thickness of the samples used for the tensile tests. 

Our results also show that both parameters were affected by changes in the crosslinker concentration and its length (Table 2). Regarding the concentration, and for the samples with three units of EG 3E-B, 3E-C, and 3ED, its increase led to a rise in the Young´s modulus and a decrease in the elongation at break. Surprisingly, the mechanical properties of Sample 3E-A are like those of 3E-B (Table 2). The elongation at break was also reduced by increasing the units of EG in the crosslinker chain up to nine units, while the twenty-three EG units showed an increased elongation (Table 2). The effect of the crosslinker length on the elastic modulus is less clear, with a lower Young´s modulus for the sample with three EG units and a higher one for one, nine, and twenty-three EG units (Table 2). 

Crosslinkers are components widely used in hydrogels to enhance their rigidity and brittleness [23,24,37,84,85]. Consequently, various authors have analyzed the effect of crosslinker concentration and length on CL mechanical properties. For instance, the study of Mohamed et al. [24] confirmed our observations regarding samples 3E-B, 3E-C, and 3E-D. These authors observed an increase in the elastic modulus of 0.4 MPa when doubling the 1-EGDMA concentration (from 0.5 at 1 wt%) in an NVP SiHy CL. Similar trends have also been observed in the work of Wu et al. [23], in which the elastic modulus increased (2.0 to 2.8 MPa), and the elongation at break decreased (178 to 85%) when the concentration of crosslinkers went from 0.5 to 1 wt%. The similar mechanical properties of samples 3E-A and 3E-B could be explained by the entanglement hypothesis previously suggested in the Dk Results and Discussion section. Indeed, more entanglements due to low crosslinking (like in sample 3E-A) could lead to a stiffening of the material [48].

Zaragoza et al. [85] also studied the effect of PEGDA length, finding that the longer the crosslinker (the higher number of EG units), the better the inside structure’s organization and, thus, the higher the elastic modulus. In contrast, Mabilleau et al. [86] found that the replacement of 3-EGDA by the longer PEGDA crosslinkers decreased the elastic moduli. These authors suggested that the chain length of the crosslinkers provided more flexibility to the material. We observed that the crosslinker length and concentration impacted CL elastic moduli in a complex way, with an increase in the modulus being observed with an increase in concentration, but when incorporated at a constant level of ~0.1 mol % (Table 3), 3-EGDMA has the minimum modulus value. The lowest elastic moduli were for sample 3E-B (3-EGDMA), but they then increased for sample 9E (9-EGDMA) and sample 23 (23-EGDMA), which reached the highest value (Table 2). Likewise, Xie et al. [87] studied the mechanical properties of polyisoprene-based vitrimer elastomers with three dicarboxylic acid crosslinkers differing in length, finding that the middle-length crosslinker provides the lowest elastic moduli. They explained that the shorter crosslinker length would constrain the chains and restrict the chain motion, increasing the elastic modulus. Conversely, the longer crosslinker chains would lead to entanglement, thus increasing the elastic moduli in both cases. 

Our results and those of other authors suggest that the concentration and length of the crosslinking agent affect the mechanical properties of contact lenses. Specifically, our results indicate that EGDMA generates softer materials and that their CL application requires the development of strategies to increase their stiffness. We tried to augment the rigidity of our samples by adding a slight amount (five wt%) of other hydrophobic monomers to the sample 3E-B, such as Dicyclopentanyl methacrylate (DCPMA) and Vinylbenzyl chloride (VBC), which are known to increase the rigidity of the material [88,89]. These modifications did not augment the Young´s moduli of the samples much (E_DCPMA_ = 0.08; E_VBC_ = 0.12) and, even worse, reduced the oxygen permeability (DK_DCPMA_ = 53 Fatt units; Dk_VBC_ = 52 Fatt units) (sample 3E-B’s properties are shown in Table 2 and Figure 1). Therefore, other strategies to enhance the rigidity of hydrogel are needed, and the introduction of nanoparticles could be one of these [20].

## 3. Conclusions

In this study, we synthesized hydrogel contact lenses based on a mixture of N,N-dimethylacrylamide, Cyclohexyl methacrylate, and different concentrations and lengths of the crosslinking agent poly (ethylene glycol) dimethacrylate. The resultant non-silicone hydrogel contact lenses presented a similar high water content and adequate properties for their clinical application, except for their wettability and rigidity, which need some improvements. Crosslinking modifications barely affected the water content, the wettability, the refractive index, the visible light transmittance, and Young’s moduli parameters of these contact lenses. In contrast, they altered the elongation at break values, oxygen permeability values, through polymer chain entanglement modifying the crosslinking level.

More importantly, the crosslinker variations provided Dk values of up to 73.90 Fatt units, approaching the permeability of silicone hydrogel contact lenses. This study highlights the crosslinker’s ability to increase the Dk value and opens new possibilities for developing more permeable non-silicone hydrogel contact lenses.

## 4. Materials and Methods

### 4.1. Materials

N,N-Dimethylacrylamide (DMAA) and Cyclohexyl methacrylate (CHMA) were purchased from Cornelius Specialities Ltd. (Haverhill, UK). Different crosslinkers were used, including ethylene glycol dimethacrylate, abbreviated as 1-EGDMA (with an average molecular weight Mw = 198 g/mol), and polyethylene glycol dimethacrylate with three, nine, and twenty-three units of ethylene glycol (EG) (abbreviated as 3-EGDMA (Mw = 286 g/mol), 9-EGDMA (Mw = 536 g/mol), and 23-EGDMA (Mw = 1136 g/mol), respectively). These crosslinkers were purchased from Sigma-Aldrich (Dorset, UK). 2,2′-Azobis(2,4-dimethylvaleronitrile), V65, was used as the polymerization initiator and was obtained from Wako Chemicals GmbH (Neuss, Germany). Phosphate buffered saline (PBS) was prepared in our laboratory using sodium phosphate dibasic from Honeywell (Bracknell, UK), sodium chloride from VWR Chemicals (Leicestershire, UK), and sodium phosphate monobasic from Sigma-Aldrich (Dorset, UK), according to ISO 18369-3:2017, 4.9 [90]. We also prepared borate buffered saline (BBS) (pH 7.2; 0.14 M).

### 4.2. Preparation of the Hydrogel Lenses

To obtain contact lenses, we employed the lathe-cutting technique. Thus, from a mixture of the hydrophilic monomer DMAA and hydrophobic monomer CHMA, the different EGDMA crosslinkers (n-EGDMA), and the initiator V65, we generated a hydrogel, using the amounts indicated in Table 3. The hydrogels were then lathe-cut in CL and finally hydrated in BBS. 

The hydrogels were formed by the free-radical polymerization method [91]. We stirred the hydrophilic and hydrophobic monomers (i.e., DMAA and CHMA, respectively) with the crosslinkers and the initiator at 20 °C for 10 min. The resultant solution was filtered in a funnel through Whatman ashless grade 40 paper (8 µm pores) from Sigma-Aldrich (Dorset, UK), degassed to remove the air contained inside the mixture, and poured into a polyethylene (PE) tube. Afterwards, we added nitrogen to the top of the tube and capped it. The mixtures were then cured in a water bath at 25 °C for 48 h and post-cured at 100 °C. Then, we extracted the resultant polymer rods from the PE tubes and cut them into buttons latched into lenses. Finally, we soaked lenses into the BBS overnight and autoclaved them at 121 °C. 

### 4.3. Characterization Methods

#### 4.3.1. Water Content

The water content (WC) of two contact lenses of each material (*n*= 2 CLs for each sample) was estimated by measuring the weight of the hydrated CL ($w_{hydrated}$) and of the same lens dried after putting it for 4 h in a vacuum oven at 80 °C ($w_{dry}$), using the following formula [35]:

(1) $$\mathrm{WC}\;(\%)=\frac{w_{hydrated}-w_{dry}}{w_{hydrated}}\;\times \;100$$

#### 4.3.2. Oxygen Permeability

Given that the thickness of the lenses is a necessary parameter to calculate the oxygen permeability, we measured it with an electronic gauge (ET-3 Rehder, Rehder Development Company, Albany, CA, USA).

We calculated the oxygen permeability (Dk) of each sample twice using a curved polarographic cell (Rehder Development Company, Albany, CA, USA) coupled to an oxygen permeameter (model 201T, Createch, Albany, CA, USA), according to the polarographic method described in ISO standard 18369-4 [35] (*n* = 4–6 CLs for each Dk value obtained). The lenses were soaked before the measurements in PBS, as suggested by ISO standard [35]. The measurements were undertaken in a closed chamber, heated with the temperature held at 35 °C, and maintained with a water-vapor-saturated atmosphere reaching at least 98% relative humidity. For the control of the Dk measurement method, we first measured the Dk of a silicone-free commercialized contact lens, Comfi Daily Disposable (Etafilcon A, Unicon Optical Co., Ltd., Taiwan). 

#### 4.3.3. Contact Angle

We measured the contact angles of two contact lenses of each material (*n* = 2 CLs for each sample) with the sessile drop technique using a drop shape analyzer (DSA25S, Krüss, Hamburg, Germany). Accordingly, after drying the surface of the CLs with a tissue, a drop of deionized water was deposited slowly on them. Then, we measured the angles during the first 2 s 20 times and took the means of the values as the contact angle. 

#### 4.3.4. Refractive Index

After soaking the synthesized CLs in BBS, refractive indexes of four contact lenses of each material (*n* = 4 CL for each sample) were measured at 589 nm using a contact lens refractometer (CLR 12-70, Index Instruments Ltd., Huntingdon, UK).

#### 4.3.5. Transmittance

To evaluate the optical transparency of the CLs, we determined the percentage transmittance (T%) of visible light (with wavelength range from 380 to 780 nm) of one hydrated lens of each material (*n* = 1 CL for each sample), using a UV-Vis spectrophotometer (Cary 60, Agilent Technologies, Santa Clara, CA, USA). 

#### 4.3.6. Tensile Test

We performed the tensile tests using a universal testing machine (Instron 3343 tensiometer, Instron, Norwood, MA, USA). First, we cut the lenses in the middle, obtaining lens strips with similar dimensions; each measured using a profile projector (V-12B, Nikon Metrology NV, Tokyo, Japan). After that, we deposited the strips in deionized water for a few seconds, reducing their dehydration before attaching them to the grips of the tensile test. Finally, we evaluated the mechanical properties of two contact lenses of each material (*n* = 2 CLs for each sample). 

## Figures and Tables

**Table 1:** Water content, light transmission, refractive index, and contact angle of the contact lens samples fabricated.

Samples	WC (%)	T (%)	n	CA (°)
Sample 1E	76.44 ± 0.19	96.00	-	89.50 ± 5.50
Sample 3E-A	80.60 ± 0.01	92.51	1.3630 ± 0.0006	92.00 ± 4.00
Sample 3E-B	80.28 ± 0.18	91.35	1.3662 ± 0.0004	100.00 ± 5.00
Sample 3E-C	78.06 ± 0.01	99.91	1.3691 ± 0.0005	86.00 ± 2.00
Sample 3E-D	75.69 ± 0.28	85.91	1.3740 ± 0.0001	81.00 ± 1.00
Sample 9E	79.15 ± 0.05	92.01	1.3691 ± 0.0000	98.50 ± 3.50
Sample 23E	78.30 ± 0.00	91.36	1.3700 ± 0.0007	97.00 ± 7.00

Results are shown as means ± standard deviation (SD). WC: Water content; T: transmittance; n: refractive index; CA: contact angle.

**Table 2:** Mechanical properties of the synthesized CLs.

Samples	Young’s Modulus (MPa)	Elongation at Break (%)
Sample 1E	0.092 ± 0.005	356.05 ± 2.35
Sample 3E-A	0.069 ± 0.008	313.45 ± 19.35
Sample 3E-B	0.066 ± 0.002	315.65 ± 13.25
Sample 3E-C	0.113 ± 0.012	178.95 ± 5.45
Sample 3E-D	0.167 ± 0.004	185.50 ± 10.50
Sample 9E	0.107 ± 0.008	243.65 ± 54.45
Sample 23E	0.120 ± 0.003	298.50 ± 36.50

**Table 3:** Formulations of the hydrogels synthesized via free-radical polymerization.

Samples	Crosslinkers (mol %; wt%)	DMAA (wt%)	CHMA (wt%)	V65 (wt%)
Sample 1E	1-EGDMA (0.10; 0.18)	79.71	20.05	0.05
Sample 3E-A	3-EGDMA (0.06, 0.15)	79.74	20.06	0.05
Sample 3E-B	3-EGDMA (0.10, 0.27)	79.64	20.04	0.05
Sample 3E-C	3-EGDMA (0.19, 0.50)	79.46	19.99	0.05
Sample 3E-D	3-EGDMA (0.38, 0.99)	79.07	19.89	0.05
Sample 9E	9-EGDMA (0.10, 0.50)	79.46	19.99	0.05
Sample 23E	23-EGDMA (0.11, 1.09)	78.99	19.87	0.05

mol %: percentage in moles of the synthesized polymer before hydration. wt%: percentage in weight of the synthesized polymer before hydration. 1-EGDMA: ethylene glycol dimethacrylate with only one ethylene glycol; 3-EGDMA: polyethylene glycol dimethacrylate with three units of ethylene glycol; 9-EGDMA: polyethylene glycol dimethacrylate with nine units of ethylene glycol; 23-EGDMA: polyethylene glycol dimethacrylate with twenty-three units of ethylene glycol; DMAA: Dimethylacrylamide; CHMA: Cyclohexyl methacrylate; V65: 2,2′-Azobis(2,4-dimethylvaleronitril).

## Data Availability

The original contributions presented in the study are included in the article, further inquiries can be directed to the corresponding author.
